# Case Report: Trilostane therapy in a dog with recurrent adrenocortical carcinoma producing an array of steroid hormones

**DOI:** 10.3389/fvets.2025.1632432

**Published:** 2025-09-10

**Authors:** Chloe Cheung, Luca Giori, Christine Griebsch, Natalie Courtman, Juan Podadera, Mary Thompson

**Affiliations:** ^1^University Veterinary Teaching Hospital Sydney, Sydney School of Veterinary Science, The University of Sydney, Camperdown, NSW, Australia; ^2^Diagnostic Endocrinology Service, Biomedical and Diagnostic Sciences, College of Veterinary Medicine, University of Tennessee, Knoxville, TN, United States; ^3^Veterinary Pathology Diagnostic Services, Sydney School of Veterinary Science, The University of Sydney, Camperdown, NSW, Australia

**Keywords:** case report, adrenocortical carcinoma, hyperaldosteronism, hyperandrogenism, trilostane

## Abstract

**Case summary:**

A 10-year-old neutered male poodle-cross was presented with signs of progressive hyporexia and marked polyuria and polydipsia (PU/PD) of 2 months' duration. Right unilateral adrenalectomy was performed 24 months prior, and adrenocortical carcinoma with no evidence of metastatic disease was diagnosed. Tumor aldosterone secretion was suspected due to persistent hypokalaemia and systemic hypertension. Upon re-presentation, the dog had a pot-bellied appearance, dermatological changes (symmetrical alopecia along the trunk, elbows, and hocks, with decubital ulcers), systemic hypertension, and marked hypokalaemia unresponsive to oral potassium supplementation, raising concerns for an endocrine disorder. Abdominal CT confirmed mass lesions in multiple liver lobes near the previous adrenalectomy site, and cytology confirmed adrenocortical carcinoma metastases. Regional and cranial mediastinal lymphadenomegaly, as well as prostatomegaly, were observed, while no abnormalities were detected in the left adrenal gland. A serum adrenal profile identified marked elevations in progesterone, androstenedione, estradiol, and testosterone concentrations pre- and post-ACTH. Serum aldosterone and cortisol concentrations pre- and post-ACTH were within reference intervals, noting the dog had been treated with spironolactone for 8 weeks at measurement. Trilostane therapy was initiated with an initial positive response, including reduced PU/PD and resolution of pot-bellied appearance. A significant reduction of steroid hormones was documented later. Signs returned about 4 months into trilostane treatment with evidence of progressive disease on repeat CT and adrenal profile. The dog is managed with palliative trilostane, 14 months since liver metastasis diagnosis.

**Relevance and novel information:**

This case highlights an initial clinical response to trilostane in a dog with metastatic, functional adrenocortical carcinoma (ACC), demonstrating short-term control of clinical signs. The variation in presentation between initial diagnosis and relapse prompted a hypothesis of a shift in tumor steroidogenic activity—a phenomenon rarely documented in veterinary literature. It underscores the diverse manifestations arising from excess production of multiple steroid hormones, including precursors. It also supports adrenal profiling in complex cases and confirms trilostane's utility as a palliative therapy in non-resectable or metastatic ACC.

## Introduction

Adrenocortical carcinoma (ACC) is an uncommon malignant tumor that arises from the cortical layer of the adrenal gland and can be functional, if overproducing steroid hormones, or can be non-functional. Clinical manifestations such as polyuria/polydipsia (PU/PD), weight gain, and variable dermatological changes frequently occur in dogs with ACC due to excessive glucocorticoid production (1). Similar clinical signs have also been observed in isolated cases where the predominant hormones produced were progesterone, corticosterone, androgens, and/or aldosterone (2–4). Treatment options for functional ACC in dogs include adrenalectomy and medical therapy to inhibit or eradicate the hormone production by the tumor, e.g., trilostane or mitotane (2, 5). In people, the reported recurrence rate for ACC after adrenalectomy was 23%, whereas the reported relapse rate was ~15% in dogs with ACC that underwent adrenalectomy (5, 6).

This case report details the excessive production of several adrenocorticosteroids from a metastatic ACC that emerged ~2 years following the surgical removal of the primary adrenal tumor. This hormonal imbalance led to the development of new clinical findings, including prostatomegaly and symmetrical alopecia. This case also documents the positive clinical and clinicopathological responses at the start of Trilostane therapy for the metastatic ACC.

## Case presentation

A 10-year-old male neutered Cavalier King Charles spaniel and poodle mix was first referred with a 5-week history of PU/PD. Body weight had increased from 12.3 kg to 13.3 kg over the preceding 5 months, with a body condition score of 6 out of 9 and no recent history of dietary change. Diagnostics revealed hyposthenuria (specific gravity of 1.004) and an unremarkable serum biochemistry profile. There were no significant findings on physical examination apart from the high body condition score. Persistent systemic hypertension was documented with an average systolic blood pressure of 180 mmHg. Venous blood gas analysis revealed mild hypokalemia (2.9 mmol/L; reference interval (RI) 3.3–4.8) and mild hypernatraemia (155 mmol/L; RI 137–150). Abdominal ultrasonographic examination detected a right adrenal gland mass (measuring 1.65 cm in width) with calcification and loss of corticomedullary definition. There was no evidence of vascular invasion. The left adrenal gland was within normal limits. A functional adrenal tumor was suspected; hence, a urine metanephrine-to-creatinine ratio was submitted to IDEXX Laboratories Pty Ltd. in New South Wales, Australia, and the result was not suggestive of pheochromocytoma (30 nmol/mmol; RI 46–255). A cortisol-secreting adrenocortical tumor was considered unlikely in the absence of associated clinicopathological changes; thus, no functional testing of the pituitary-adrenal axis was performed. An aldosterone-secreting adrenocortical tumor was suspected in light of hyposthenuria, electrolyte abnormalities, and systemic hypertension. A head, thoracic, and abdominal CT scan was performed to characterize the right adrenal mass further and to assess for metastatic disease. The mass measured ~1.9 cm in width × 2.8 cm in height × 2.4 cm in length and was hypodense and mildly contrast-enhancing. It was ovoid with a thin linear mineralization at the center of the mass. The mass was in contact with the caudal vena cava, causing moderate to marked extraluminal compression, but there was no overt sign of vascular invasion. Unilateral adrenalectomy was scheduled for the following week. Oral administration of spironolactone, an aldosterone antagonist (1 mg/kg PO q12h), was commenced, as was amlodipine (0.2 mg/kg PO q24h) to manage hypertension. On the day of surgery, hypokalemia was no longer evident on blood gas analysis (3.4 mmol/L; RI 3.3–4.8), but hypertension persisted (180 mmHg). Hydrocortisone (0.5 mg/kg/h) was administered intravenously intra- and post-operatively.

**Figure 1:**
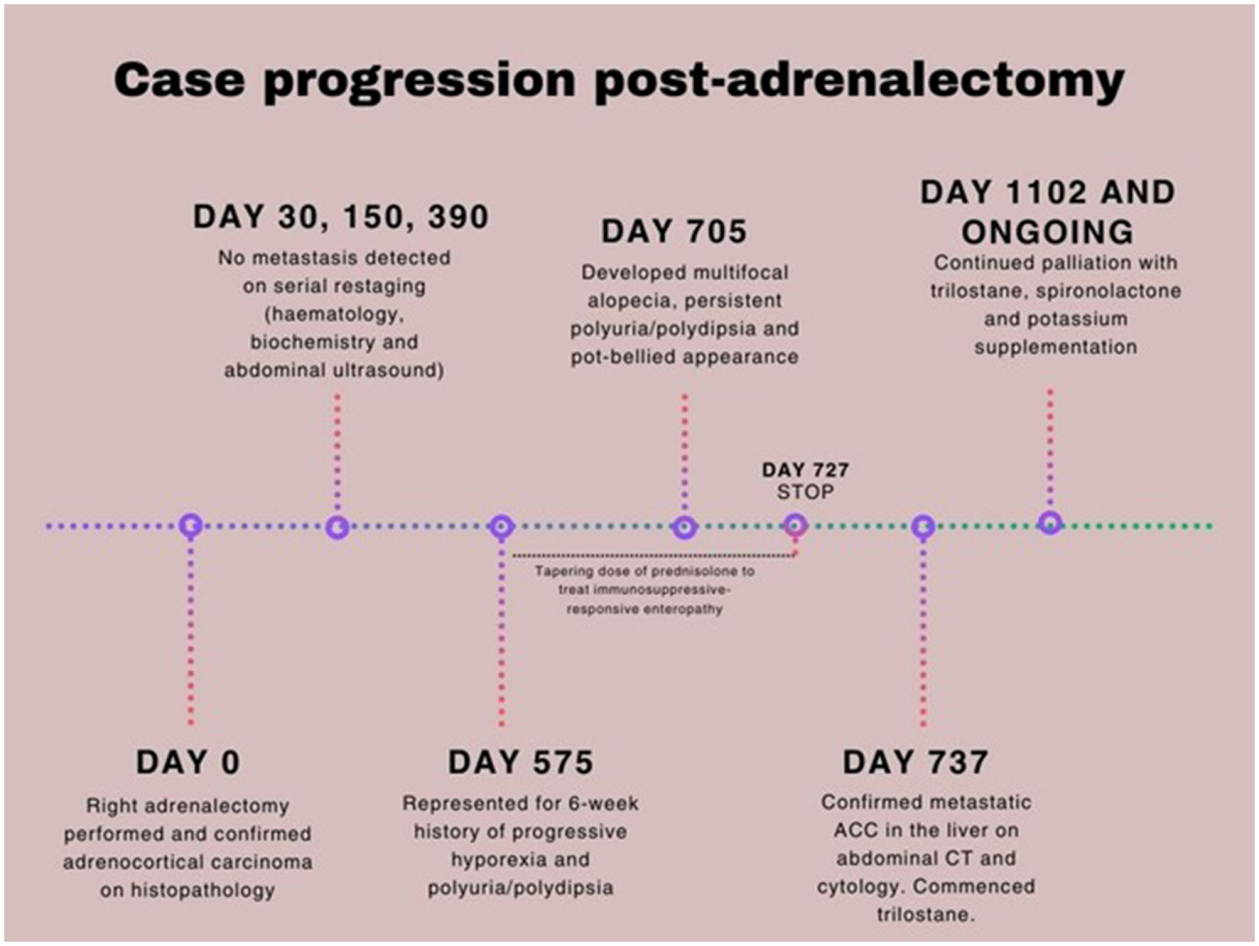
Case timeline of metastatic adrenocortical carcinoma in a 10-year-old neutered male poodle cross.

Adrenalectomy was performed without complication, and the patient recovered uneventfully. Histopathology revealed neuroendocrine cells with variably distinct cell margins, moderate amounts of finely granular eosinophilic cytoplasm, and centrally to basally located round to ovoid nuclei containing coarse chromatin and variably distinct, singular central nucleoli. There was mild anisocytosis and anisokaryosis, with one mitotic figure per 10 high-power fields. Immunohistochemistry using chromogranin A and S100 confirmed the diagnosis of ACC. Follow-up visits in the post-operative period showed resolution of hypokalemia, hypernatremia, an increase in urine specific gravity (USG 1.040), and normalization of blood pressure. Thoracic radiographic and abdominal ultrasound examinations performed at 30, 150, and 390 days after surgery showed no evidence of recurrent local or metastatic disease (Figure 1).

On Day 575 post-adrenalectomy, the dog was presented with a 6-week history of progressive hyporexia and PU/PD (Figure 1). Body weight had decreased from 11.8 kg to 10.6 kg in the preceding 4 weeks. Physical examination was unremarkable. Complete blood count and serum biochemistry profile were within reference intervals. Serum C-reactive protein concentration was mildly elevated (38.10 mg/L; RI 0.0–10.7). Urinalysis revealed a specific gravity of 1.015. Abdominal ultrasound showed an enlarged and hyperechoic pancreas, most suggestive of chronic pancreatitis. A low-fat prescription diet was recommended. The dog was readmitted on Day 635 post-surgery for persistent hyporexia. Venous blood gas analysis showed mild hypokalemia (2.9 mmol/L; RI: 3.3–4.8) and marginal hypernatremia (151 mmol/L; RI: 137–150). Potassium gluconate (4 mmol PO q12h) was supplemented. Hypertension had worsened (systolic blood pressure 220 mmHg). Given the history of functional ACC, an ACTH stimulation test was performed, and the post-stimulation cortisol concentration was within reference intervals. At this stage, chronic enteropathy was considered a possible differential diagnosis for persistent hyporexia. Hematology and biochemistry were unremarkable. Hypocobalaminaemia (196 μg/L; RI: 0–200) was suggestive of distal small intestinal disease and was addressed with cobalamin supplementation. This later prompted a gastroduodenoscopy and colonoscopy, which revealed no significant abnormalities on direct visual examination of the mucosa. Histopathology showed mild to moderate lymphoplasmacytic and eosinophilic enteritis. This finding suggested gastrointestinal potassium loss as the more likely cause, rather than renal loss, especially since the urine fractional excretion of potassium was within normal limits. A gradual transition to a strict hydrolyzed diet and oral prednisolone administration (2 mg/kg/day in divided doses) were commenced (Figure 1). The prednisolone dose was reduced by ~25% every 2–3 weeks based on positive clinical responses, including gradual weight gain and improved appetite.

**Figure 2:**
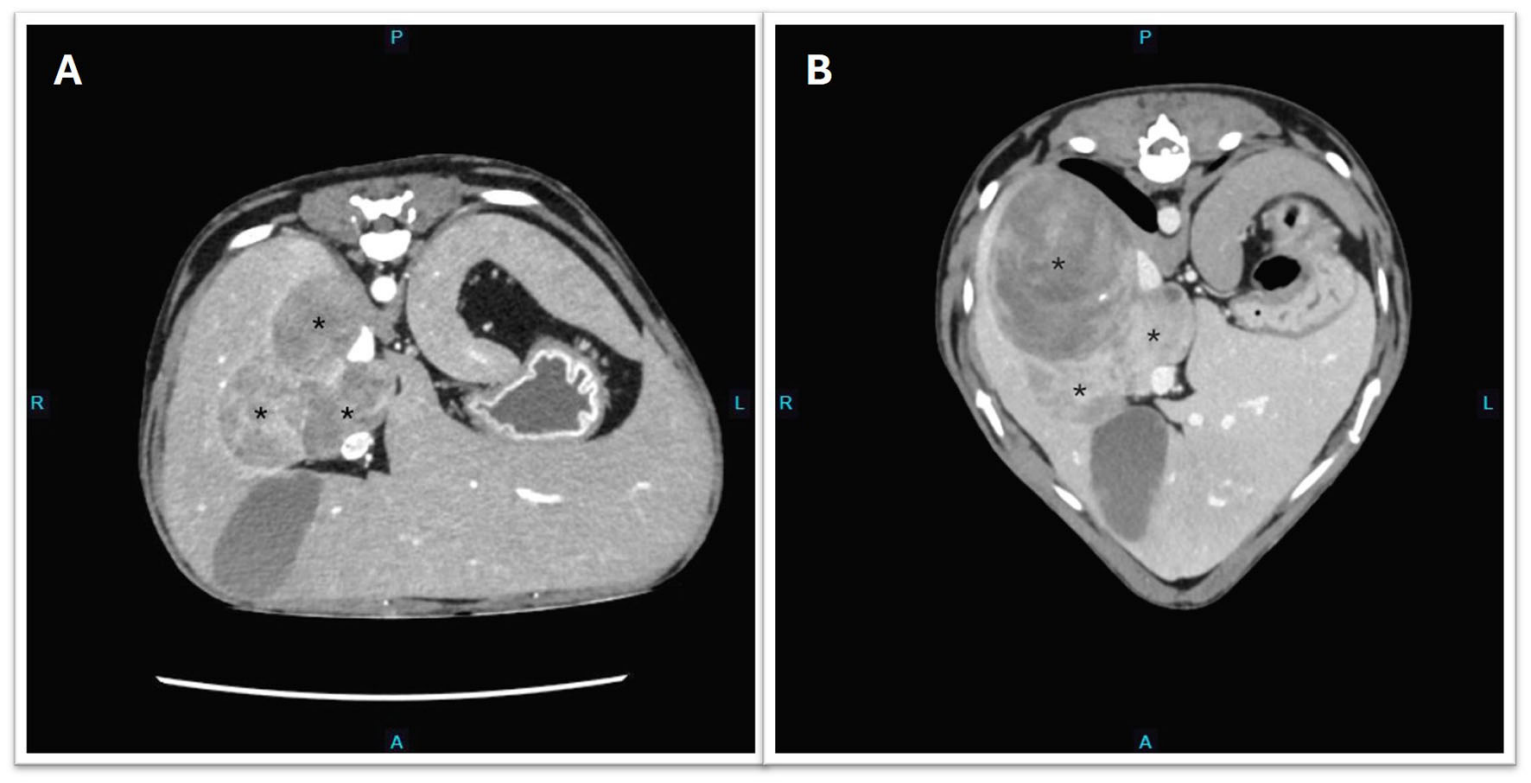
Transverse CT images showing the progression of metastatic hepatic masses (depicted by asterisk*). **(A)** Day 737 and before initiation of trilostane. **(B)** Day 947 post-adrenalectomy and the dog was treated with trilostane for 7 months.

**Figure 3:**
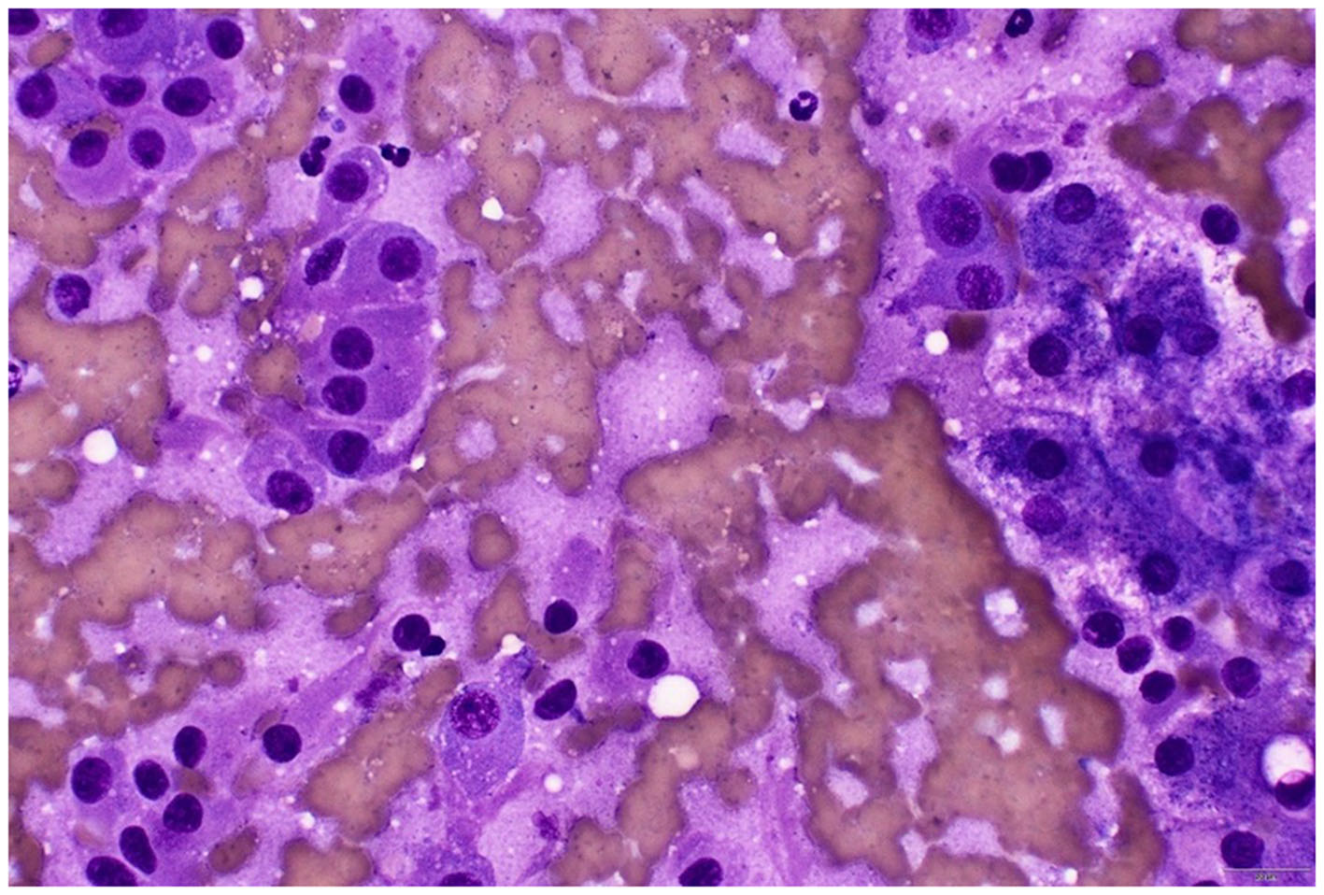
Cytology of the hepatic mass (rapid Romanowsky stain). To the left of the image are the ACC cells with eccentric nuclei with no distinct nucleoli, and to the right of the image are hepatocytes with vacuolar changes, likely from glycogen accumulation secondary to the functional ACC. Naked nuclei are evident in the background. Lower right scale bar is 20 μm.

By Day 705 (2 months into the treatment of chronic enteropathy), the dog had developed bilateral symmetrical alopecia over the ventrum, elbows, hocks, and tail, accompanied by significant PU/PD and a pot-bellied appearance (Supplementary Figure 1). The dog had begun to display mounting and aggressive behavior toward his littermate. Despite ongoing potassium supplementation, moderate hypokalemia (2.7 mmol/L; RI 3.6–5.6) was documented on Day 720 on serum biochemistry, which also revealed a moderate degree of cholangiohepatopathy with significantly increased ALT (709 U/L; RI 10–95) and ALP (534 U/L; RI 0–94) activities, findings not previously observed. While prednisolone tapering continued until discontinuation on Day 727, iatrogenic hyperadrenocorticism was initially considered. Nonetheless, the dog persisted in being severely polyuric and polydipsic with unresolved dermatological lesions 2 weeks after prednisolone discontinuation. Given the history of ACC and increasing clinical signs of hyperadrenocorticism, metastatic disease was suspected. A head, thoracic, and abdominal CT scan was pursued on Day 737 (Figure 2). Three hypoattenuating heterogenous contrast-enhancing masses in the right hepatic lobe with regional lymphadenopathy were identified. The left adrenal gland was unremarkable. The prostate was moderately enlarged and smoothly marginated (2.9 cm wide) with normal periprostatic tissues. The bladder was markedly distended. The pituitary gland was unremarkable on head CT. Ultrasound-guided aspirations of the hepatic masses and “normal” hepatic tissue were performed. Cytology of the hepatic masses revealed epithelial cells with round nuclei containing fine to lacy chromatin, indistinct nucleoli, and moderate amounts of purple cytoplasm, often with fine vacuoles. The cells exhibited moderate anisokaryosis and anisocytosis, with occasional binucleation and mitoses. These cytomorphological features were consistent with malignancy and strongly suggestive of metastatic ACC. In contrast, the normal hepatocytes showed changes consistent with vacuolar hepatopathy (Figure 3).

Given the behavioral changes and new imaging findings of prostatomegaly, concern around the possibility of secretion of multiple steroid hormones from metastatic ACC prompted further investigation. A serum canine-validated adrenal profile was submitted to the University of Tennessee on Day 737. It showed marked elevations in several intermediate steroids, including androgens (i.e., androstenedione and testosterone), estradiol, and progesterone. Both cortisol and aldosterone concentrations were within reference intervals before and after the ACTH stimulation test (Table 1).

**Table 1:** Adrenal profile before trilostane, 14 days, 7 months, and 13 months into trilostane treatment.

**Test**	**Baseline before treatment**	**Baseline at 14 days**	**Baseline at 7 months**	**Baseline at 13 months**	**Normal RI (baseline)**	**Post-ACTH before treatment**	**Post-ACTH at 14 days**	**Post-ACTH at 7 months**	**Post-ACTH at 13 months**	**Normal RI (post-ACTH)**
Cortisol (μg/dL)	5.2	1.7	1.5	1.8	< 1.0–5.6	8.4	1.7	2.9	3.7	7.1–15.1
Androstenedione (ng/mL)	>10.0	>10.0	>10.0	>10.0	0.05–0.36	>10.0	>10.0	>10.0	>10.0	0.24–2.90
Estradiol (pg/mL)	208.7	111.4	58.3	41.3	23.1–65.1	207.8	110	76.9	60.6	23.3–69.4
Progesterone (ng/mL)	11.4	3.47	3.85	5.9	< 0.20	>40.0	4.42	25.4	34.6	0.22–1.45
17-OH-progesterone (ng/mL)	< 0.08	14.72	>25.0	>25.0	0.08–0.22	< 0.08	20.32	>25.0	>25.0	0.25–2.63
Testosterone (ng/dL)	1,568	358	618	1,589	< 15.0–24.0	>1,600	272	830	>1600	< 15.0–42.0
Aldosterone (pg/mL)	46.8	9.3	45.2	39.2	6.7–253.6	202.7	11	90.2	91.3	55.6–737.2

Shaded areas showed abnormal results.

Treatment options, including surgical resection, medical therapy with trilostane, and o,p'-DDD (mitotane), with or without adjunctive chemotherapy, were discussed with the owners. Trilostane (2.5 mg/kg PO q12hr) was subsequently commenced, and a repeat steroid profile 2 weeks into treatment showed significant reductions in estradiol, progesterone, and testosterone. However, a significant elevation in 17-hydroxyprogesterone concentration was noted (Table 1). Post-ACTH cortisol and aldosterone concentrations were both within reference intervals. The dog was reportedly more energetic, with less pronounced PU/PD, and his pot-bellied appearance improved progressively (Supplementary Figure 1). Potassium concentration was stable (4.3 mmol/L; RI 3.3–4.8) on cessation of spironolactone, and urine was concentrated (USG 1.042). Four months after initiating trilostane treatment, the dog developed recurrent clinical signs, including PU/PD, a pot-bellied appearance, and mounting behavior toward the littermate. Progressive disease was confirmed on a repeat abdominal CT performed on Day 947 with an evident increase in size and volume of the dorsally located hepatic mass (Figure 2). A repeat adrenal profile also documented persistent disarray of intermediate steroid hormones (androstenedione, estradiol, progesterone, 17-hydroxyprogesterone) and an increase in testosterone concentration (Table 1). The suboptimal control of clinical signs and documentation of rising hormones prompted an increase in trilostane dose (5 mg/kg PO q12hr). The cortisol concentration was within a safe range for the increase in dosing. At the time of writing, the dog had been receiving trilostane for 14 months, 420 days after the diagnosis of the liver metastasis, and 1,275 days since adrenalectomy. The owners elected to continue with palliative management with trilostane, spironolactone, amlodipine, and potassium supplementation, albeit with persistent clinical signs.

## Discussion

This case illustrates an initial clinical and clinicopathological response to trilostane in a dog with metastatic ACC. Trilostane therapy resulted in a notable reduction in serum concentrations of multiple steroid hormones, including estradiol, testosterone, and progesterone, with concurrent clinical improvement. The resolution of PU/PD, improved energy, and reversal of pot-bellied appearance demonstrate that palliative medical management can provide short-term benefit in cases where surgical or cytotoxic options are not pursued. While trilostane is typically used to control diseases that result in excessive cortisol release, this case supports its broader utility in suppressing the clinical effects of hormonally active adrenal tumors. Previous reports have described the use of trilostane in similar contexts, with most demonstrating favorable initial responses (7–9). One case report documented cytoreduction of a metastatic ACC and clinical improvement in a Maltese dog treated with trilostane, which survived over 1 year and later succumbed to surgical complications from cholecystectomy (9). Although cytoreduction was not observed in this case, the dog has enjoyed a longer survival time (420 days beyond the diagnosis of metastasis and still alive) compared to the reported case using combined treatment of palliative trilostane, spironolactone, amlodipine, and potassium supplementation. The owners' primary goal was to preserve their dog's comfort and quality of life following the diagnosis of metastatic disease. While alternative options such as surgical debulking or a transition to mitotane therapy were discussed, the owners opted against pursuing more aggressive interventions. Their decision was influenced by a desire to avoid heroic measures and the practical limitation that mitotane is only available in capsule form in Australia, which they found difficult to administer. Given the dog's otherwise stable condition and prior positive response to trilostane, they elected to continue palliative management with this therapy. The owners reported modest improvements in the dog's demeanor and resolution of some dermatological and behavioral signs. They have continued to monitor the dog's wellbeing and expressed satisfaction with the overall outcome, noting that the dog has remained interactive and comfortable for over a year following the diagnosis of metastasis.

The evolution in clinical signs between initial diagnosis and relapse suggested a potential expansion of the classes and/or alterations in the ratios of steroid hormones produced by a metastatic functional ACC in a dog. At the initial presentation, the dog exhibited signs suggestive of hyperaldosteronism, including persistent hypokalaemia that was non-responsive to supplementation, and systemic hypertension. Notably, no dermatological, prostatic, or behavioral changes were observed at that time. In contrast, the relapse was characterized by overtly sexualized behavior, prostatomegaly, and symmetrical alopecia, accompanied by elevated testosterone and estradiol concentrations (Table 1). This variation raises the possibility of a transition in the tumor's steroidogenic profile over time, potentially due to tumor heterogeneity or altered enzyme expression within the metastatic lesion. Such a transition has been speculated in human oncology but has not been reported in veterinary medicine, making this case particularly noteworthy. Co-production of multiple steroid hormones in human ACC is also rare (10). A recent case report in a Labrador retriever described hypersecretion of progesterone at both initial presentation and at metastatic relapse with a similar timeline to the current case—~2 years after the initial surgery (3).

Hyperaldosteronism is rare in dogs compared to cats, with polyuria and general weakness being the most reported presenting signs, likely due to electrolyte derangement and volume depletion (11). While spontaneous mineralocorticoid overproduction by an adrenocortical tumor itself is one recognized mechanism, an alternative explanation described in human ACCs involves mineralocorticoid receptors hyperactivated by excessive cortisol, leading to persistent hypertension and hypokalaemia (11). Excessive cortisol secretion may have contributed to the PU/PD, weight gain, and clinical signs mimicking hyperaldosteronism in the current case, potentially through cortisol-mediated activation of the mineralocorticoid receptor. At re-presentation, the dog exhibited clinical signs suggestive of hypercortisolism, yet cortisol concentrations remained within reference intervals. Similar discrepancies have been reported in both veterinary and human medicine; overt clinical signs of hyperadrenocorticism occur in the absence of confirmatory diagnostic results (12, 13). Disorganized steroidogenesis is a recognized phenomenon in functional ACC in humans, which is characterized by irregular expressions of the steroidogenic enzymes involved in hormone synthesis, and this leads to unregulated steroid hormone production, which may also contribute to the inability to detect altered steroid molecules in standard assays due to their molecular disarray (14, 15). This may be one of the contributing factors to the normal cortisol concentration documented at re-presentation. Additionally, high concentrations of progesterone may suppress the hypothalamic-pituitary-adrenal axis, leading to physiologically low cortisol concentrations despite ongoing clinical signs (16). This mechanism is suggested in other reports of dogs and cats with functional adrenal tumors producing primarily sex or intermediate steroids (2, 3, 16).

To the authors' knowledge, there is currently no literature directly addressing the prognostic implications of performing adrenal function testing before adrenalectomy in dogs. In cats with adrenal tumors performing an adrenal profile as part of the preoperative work-up has been recommended however as hypercortisolism and the secretion of multiple steroid hormones have been documented, potentially influencing the peri- and post-operative management in this species (17, 18). A major limitation in this case was that complete adrenal profile was not performed at the time of the initial presentation. As a result, direct comparison of the tumor's steroidogenic output at initial presentation and relapse was not possible, precluding confirmation of a shift or expansion in hormone production over time. This limitation was due to the unavailability of a validated canine-specific adrenal profile in Australia and a desire to proceed with surgical resection of the adrenal mass in a timely fashion. Future documentation of adrenal hormones in dogs with suspected ACC at initial presentation could help identify transitions in steroid hormone production, particularly in cases that re-present with different clinical signs.

Another limitation in the diagnostic evaluation of this case was the lack of measurement of endogenous ACTH concentration, which precluded the exclusion of ACTH-dependent hyperadrenocorticism as a differential diagnosis. While the observed clinical signs and markedly elevated sex hormone concentrations supported the diagnosis of a functional ACC, the normal serum cortisol concentration raised the possibility of pituitary-dependent hyperadrenocorticism or, less commonly, ectopic ACTH production, despite a normal pituitary on the head CT. In humans, Ectopic ACTH secretion from epithelial neoplasms, such as pulmonary and neuroendocrine tumors, has been described as a cause of ACTH-dependent Cushing's syndrome (19). More relevantly, *de novo* ACTH synthesis by a primary ACC has also been reported in human patients, particularly those with high-grade malignancy, resulting in clinical hypercortisolaemia despite an adrenal origin (20). In our case, without ACTH testing, we cannot fully exclude the possibility of concurrent or ectopic ACTH production, nor can we definitively distinguish between ACTH-dependent and independent disease. This highlights the importance of incorporating endogenous ACTH measurement in the diagnostic work-up of adrenal mass, particularly in cases where cortisol concentrations are equivocal.

The dog in this case demonstrated elevated serum 17-hydroxyprogesterone concentrations at 14 days (20.32 ng/mL; RI: 0.08–0.22) and 7 months (>25.0 ng/mL) following the initiation of trilostane therapy (Table 1). Baseline 17-hydroxyprogesterone concentration was < 0.08 ng/mL. Trilostane inhibits 3β-hydroxysteroid dehydrogenase, an enzyme responsible for converting pregnenolone into progesterone and its downstream precursors such as 17-hydroxyprogesterone and androstenedione. While inhibition of this pathway would theoretically reduce concentrations of these precursors, paradoxical increases are frequently observed during trilostane treatment (21). The accumulation of these intermediate steroid precursors has been associated with persistent clinical signs of hyperadrenocorticism despite optimized cortisol concentrations (21). One possible explanation is the dysregulated steroidogenesis characteristic of poorly differentiated or immature ACC cells, which may lead to the accumulation of precursor steroids and metabolites rather than end products, such as cortisol or aldosterone (22). In human medicine, this metabolic complexity has led to the development of serum and urine metabolomics to overcome the limitations of conventional hormone assays in monitoring ACC activity. Liquid chromatography–tandem mass spectrometry (LC-MS/MS) has become the preferred method for steroid metabolite analysis due to its superior specificity and accuracy compared to traditional immunoassays (23). However, such techniques are not yet routinely applied in veterinary endocrinology, which limits the ability to characterize atypical steroidogenic profiles in dogs with functional adrenal tumors.

Overtly sexualized behaviors—such as increased aggression, mounting, and excessive vocalization have been reported in cats with sex hormone-producing functional adrenal tumors (4). In this case, the serum testosterone concentration was 1,568 ng/dL (RI: < 15–24) before trilostane treatment, much higher than the reported median concentration for intact male dogs of 280 ng/dL (24). Recurrent mounting behavior was noted to coincide with the rising testosterone concentration on repeat adrenal profile (Table 1). These findings suggest that severely elevated testosterone concentrations were the most likely driver of the behavioral changes and prostatomegaly in this case.

There was an ~5-month lag from re-presenting clinical signs to detecting metastasis on imaging. An abdominal ultrasound, performed by a board-certified radiologist during the initial re-presentation period, failed to detect any metastatic lesions. Abdominal CT has been shown to have a superior detection rate over abdominal ultrasound in regional lymphadenomegaly in a cohort of dogs with apocrine gland adrenocarcinoma (25). This case highlights the importance of adrenal function testing in cases where a relapse of clinical signs occurs, irrespective of imaging findings on abdominal ultrasound. Abdominal CT should be considered, when available, over abdominal ultrasound for metastatic screening in dogs with relapse of clinical signs.

## Take home messages

In cases of non-resectable functional ACC, trilostane may be utilized for palliation, providing partial clinical resolution. It is crucial to perform adrenal profiling in conjunction with diagnostic imaging in patients with supportive clinical features of adrenal hypersecretion that have undergone adrenalectomy, as this combination enhances diagnostic accuracy. Notably, normal cortisol and aldosterone concentrations can still be observed in functional ACC due to molecular derangements in steroid hormone synthesis, which may interfere with assay performance. Therefore, a comprehensive approach that integrates clinical signs, laboratory results, imaging findings, and adrenal profiles is essential for accurate interpretation and diagnosis in suspected cases of functional ACC.

## Data Availability

The original contributions presented in the study are included in the article/Supplementary material, further inquiries can be directed to the corresponding author.

## Supplementary material

Supplementary Figure 1 **(A)** Dermatological changes and pot-bellied appearance of the dog on Day 705 (left). **(B)** Same dog showing improved skin changes and pot-bellied appearance 2 weeks after Trilostane therapy (right).
