# Supplementary figures and images for: Case Report: Trilostane therapy in a dog with recurrent adrenocortical carcinoma producing an array of steroid hormones
Source: Front Vet Sci. 2025 Sep 10;12:1632432. doi: 10.3389/fvets.2025.1632432 (PMC12458880; doi:10.3389/fvets.2025.1632432)

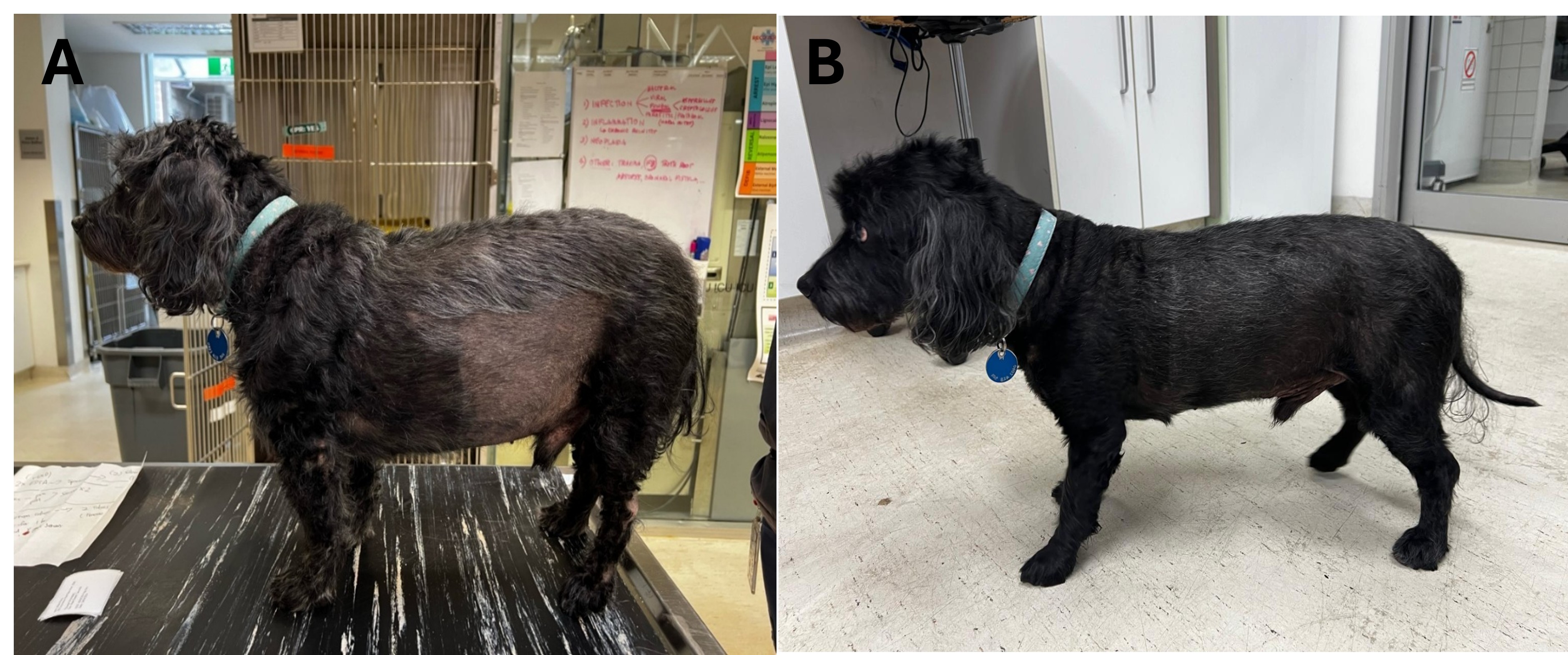

Supplement: Supplementary Figure 1 — (A) Dermatological changes and pot-bellied appearance of the dog on Day 705 (left). (B) Same dog showing improved skin changes and pot-bellied appearance 2 weeks after Trilostane therapy (right). [file Image_1.jpeg]
